# Evaluation de la formation des résidents en chirurgie générale et digestive en Tunisie

**DOI:** 10.11604/pamj.2015.21.328.6604

**Published:** 2015-08-31

**Authors:** Ammar Mahmoudi, Faouzi Noomen, Mohamed Nasr, Khadija Zouari, Abdelaziz Hamdi

**Affiliations:** 1 Service de Chirurgie Générale et Digestive, CHU Fattouma Bourguiba de Monastir, Tunisie

**Keywords:** Résidanat, chirurgie, pédagogie médicale, formation, recherche, Residency, surgery, medical education, training, research

## Abstract

**Introduction:**

De nombreux moyens sont mis à disposition des résidents en chirurgie générale et digestive pour assurer leur formation théorique et pratique. Cependant, le niveau d'utilisation de ces différents outils et leur impact sur la formation des résidents n'ont jamais été évalués. L'objectif de notre étude était d’étudier l’état des lieux des moyens de formation utilisés par les résidents pour évaluer leurs degrés de satisfaction et leurs propositions en vue d'améliorer leur formation.

**Méthodes:**

Un questionnaire anonyme a été distribué aux résidents de chirurgie générale et digestive de l'année 2012-2013. Ce questionnaire portait sur les caractéristiques démographiques, les ressources pédagogiques, ainsi que le cursus médical et universitaire. Une évaluation de la formation ainsi qu'un recueil des propositions faites en vue d'améliorer leurs formations étaient réalisées.

**Résultats:**

Cinquante résidents sur 83 ont répondu au questionnaire. L'orientation de carrière la plus fréquente était l'hospitalo-universitaire dans 70% des cas. La pratique quotidienne et l'internet étaient les deux ressources pédagogiques les plus utilisées. La formation chirurgicale était jugée satisfaisante par seulement 10% des répondants. Parmi l'ensemble des propositions faites, l'apprentissage sur simulateur chirurgical, l'existence d'un ouvrage national de référence, et l'institution d'un tutorat par un chirurgien senior recueillaient plus de 80% d'avis favorable.

**Conclusion:**

La majorité des résidents jugent leur formation non satisfaisante. Une meilleure information sur les ressources déjà existantes, un renforcement du compagnonnage et un accès plus large à un apprentissage sur simulateur chirurgical permettraient de diminuer ce sentiment d'insatisfaction.

## Introduction

En Tunisie, de nombreux moyens pédagogiques sont mis, actuellement, à la disposition des résidents en chirurgie générale et digestive pour assurer leur formation. Le cursus de chirurgie générale et digestive repose en effet sur deux types de formation, à savoir l'enseignement facultaire (cours de collège de spécialité) et la formation pratique hospitalière au bloc opératoire et au lit du malade. Cependant, l'utilisation de ces différents moyens et leur impact respectif sur la formation des résidents n'ont jamais été évalués. L'objectif de cette étude était d’étudier l’état des lieux des moyens utilisés par les résidents de chirurgie générale et digestive pour leur formation théorique et pratique et d’évaluer leurs degrés de satisfaction et leurs propositions en vue d'améliorer leur formation.

## Méthodes

Nous avons distribué un questionnaire anonyme entre le 15 mars 2013 et le 15 novembre 2013 (deux relances) aux 83 résidents de chirurgie générale et digestive. La liste des résidents a été récupérée auprès des secrétaires des services de chirurgie générale et digestive des différents centres hospitalo-universitaires (CHU) de la Tunisie. Le questionnaire comprenait 21 sections avec des questions portant sur les caractéristiques démographiques, les prérequis avant de débuter le résidanat, l´avancée dans le cursus médical et les projets post-résidanat, les ressources pédagogiques utilisées au quotidien au cours du résidanat pour la formation théorique et pratique, l´accès aux formations régionales et nationales, la réalisation d´un stage inter-CHU, le travail universitaire, le recours à un certificat d’étude complémentaire (CEC), l´appréciation de la formation théorique et pratique et les propositions en vue d'améliorer leur formation. Le questionnaire anonyme était récupéré auprès des résidents. Le but de cette étude était précisé aux répondants. Les résultats des questionnaires sont présentés pour les variables quantitatives sous la forme de moyennes. Les variables nominales étaient représentées sous la forme de pourcentages en fonction des effectifs. L´analyse statistique a été réalisée à l´aide du logiciel Excel.

## Résultats

**Caractéristiques générales**: en 2012/2013, le nombre de résidents de chirurgie générale et digestive en Tunisie était de 83. Au total, 50 questionnaires ont été analysés soit 60,22% de la population sollicitée. Les caractéristiques générales de la population d´étude sont présentées dans le Tableau 1. La moyenne d´âge de la population de l´étude était de 28,7 ans, avec un sexe ratio de 1,9 homme pour une femme. L'ancienneté moyenne des résidents était de trois semestres.

**Tableau 1:** Caractéristiques générales de la population d’étude

Caractéristiques générales		Nombre pourcentage (%)
Age, moyenne		28,7
**Sexe**	Homme	33 (66)
	Femme	17 (34)
**Faculté d'origine**	Monastir	9 (18)
	Sfax	7 (14)
	Sousse	10 (20)
	Tunis	24 (48)
**Orientation de carrière**	Hospitalo- universitaire	35 (70)
	Hospitalier	6 (12)
	Libéral	6 (12)
	Non encore décidé	3 (6)

**Parcours professionnel:** La moitié des répondants (50%) avaient choisi la chirurgie générale et digestive pour l'attractivité de la spécialité, 22 résidents (44%) pour des raisons de classement au concours de résidanat qui empêchait de choisir la spécialité voulue, et seulement 3 résidents (6%) pour des raisons financières. 32 résidents (64%) avaient débuté le résidanat dans leur région d´origine. Parmi ceux ayant débuté en dehors de la région d´origine, 44,5% ont quitté leur région d´origine pour des raisons personnelles (raisons familiales), 44,5% ont choisi de quitter leur région du fait de l´attractivité professionnelle de la région d´accueil et 11% pour des raisons de classement au concours de résidanat. Le nombre de résident en formation était jugé en adéquation avec le nombre de postes d'assistant hospitalo-universiataire (AHU) proposés dans seulement 18% des cas. Dans 82% des cas, le nombre de postes d'AHU proposés était jugé trop faible par rapport au nombre de résident en formation. L'orientation de carrière la plus fréquente était l'Hospitalo-Universitaire dans 70%, l'orientation vers le secteur libéral était notée dans 6 cas (12%), et vers le secteur hospitalier dans 6 cas (12%). Seulement 3 résidents (6%) ne savaient pas encore vers quelle carrière ils allaient s'orienter.

**Prérequis**: Avant de débuter le résidanat en chirurgie, 32% (n = 16) des répondants considéraient que la formation en anatomie était insuffisante. 60% (n = 30) des résidents jugeaient la connaissance en anatomie partiellement suffisante pour débuter le résidanat en chirurgie. Seulement cinq (10%) des personnes interrogées ont eu recours à une formation en anatomie complémentaire (CEC, dissection en laboratoire d'anatomie), avec un bénéfice en terme d´acquisition de connaissances ressenti dans 100% des cas. Une formation spécifique à la chirurgie en début de résidanat est jugée utile par 92% des répondants, mais seulement 22% (N = 11) ont pu en bénéficier. Hormis les cours théoriques, les deux types d´enseignement les plus fréquents étaient une formation chirurgicale sur cadavre (n = 8, 73%) ou sur animal (n = 5, 45,5%).

**Figure 1:**
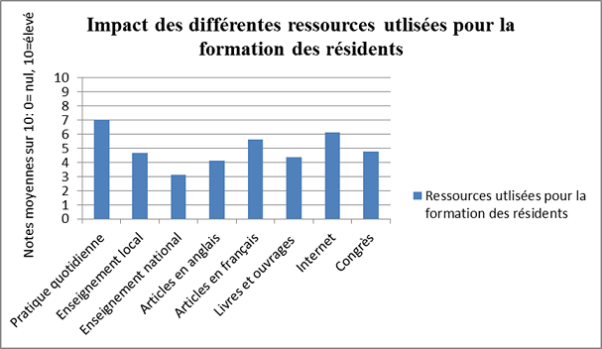
Impact des différentes ressources proposées dans la formation théorique au cours du résidanat

**Formation théorique au cours du résidanat**: L´impact des ressources utilisées pour la formation théorique est présenté sur la Figure 1. La pratique quotidienne (visites, staff médico-chirurgical et réunions de bibliographie) ainsi que l'internet étaient les deux ressources les mieux notées (7,05/10 et 6,55/10), tandis que les cours du collège de chirurgie à l’échelle nationale étaient les plus mal notés (3,77/10). Les répondants étaient libérés de leurs activités hospitalières par les chefs de services pour accéder aux cours du collège dans 36% et aux congrès dans 44%. Le mode de financement pour s´y rendre était des fonds personnels dans 74% des cas, et une prise en charge par des laboratoires dans 30% des cas.

**Figure 2:**
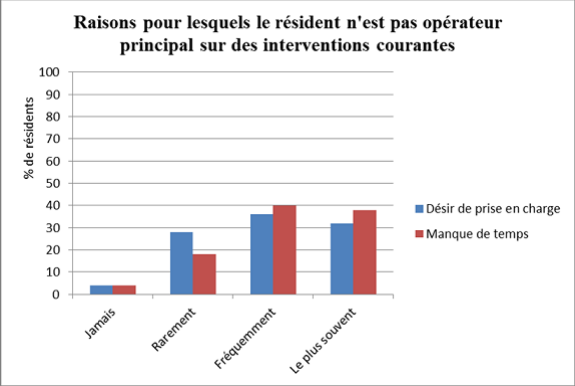
Raisons pour lesquelles le résident n'est pas opérateur principal sur des interventions courantes (hernie, vésicule, éventration)

**Formation pratique en chirurgie**: Les données concernant la formation pratique au cours du résidanat sont synthétisées dans le Tableau 2. Selon les résidents interrogés, la qualité de la formation pratique en chirurgie au cours du résidanat dépendait par ordre décroissant du nombre d´intervention en premier opérateur aidé (8,2/10), du nombre d´interventions pratiquées en premier opérateur seul (7,8/10), puis du nombre d´interventions auxquelles ils ont assisté en tant qu´aide (7,3/10). Selon les répondants, la formation pratique chirurgicale était assurée au quotidien par les AHU dans 28% des cas, par les professeurs agrégés (Pr Ag) dans 14% des cas et par l´ensemble des chirurgiens seniors sans distinction dans 66% des cas. 31 des répondants (62%) jugeaient que dans les services où ils étaient passés, il n´existait pas de politique visant à laisser opérer les résidents. Plusieurs propositions concernant les raisons expliquant qu´on ne laisse pas le résident être l´opérateur principal sur une intervention courante (vésicule, hernie, éventration) étaient faites. Les résultats sont détaillés dans la Figure 2. Le manque de temps et le désir du sénior de prendre en charge lui-même le malade étaient majoritairement évoqués (>65%) tandis que la pression médico-légale n´était que rarement évoquée. L´accès au cours du résidanat à un simulateur chirurgical ou à un apprentissage chirurgical sur l´animal était rapporté par 13 des résidents (26%), alors que des séances de dissection sur cadavre étaient rapportées par 2 répondants (4%).

**Tableau 2:** Formation pratique au cours du résidanat

Formation pratique		N = 50(%)
Politique des services visant à laisser les résidents opérer	Oui	19 (38)
	Non	31 (62)
Simulateur chirurgical	Oui	11 (22)
	Non	39 (78)
Chirurgie sur animal	Oui	2 (4)
	Non	48 (96)
Chirurgie sur cadavre	Oui	2 (4)
	Non	48 (96)

**Figure 3:**
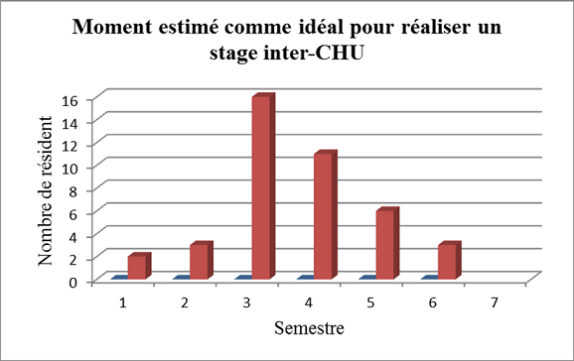
Moment estimé comme idéal pour faire un stage inter-CHU

**Formations complémentaires au cours du résidanat**: Les données concernant les formations complémentaires au cours du résidanat sont synthétisées dans le Tableau 3. **Stage inter-CHU (dans un CHU autre que les CHU liés à la faculté d'origine)**: Parmi les résidents interrogés, 44 (88%) considéraient que la réalisation d´un stage inter-CHU participait à l´amélioration de la formation chirurgicale. Le moment idéal estimé pour réaliser ce stage au cours du cursus était le 3ème ou le 4ème semestre pour la plupart des personnes interrogées (Figure 3). 56% des résidents avaient déjà réalisé ou envisageaient de réaliser un stage inter-CHU. Les raisons de ce stage inter-CHU étaient essentiellement l´acquisition d´une compétence (40%) et la recherche d´un poste d'AHU (8%). Lorsqu´il avait été réalisé, ce stage était jugé bénéfique sur le plan théorique et pratique dans respectivement 91,6% et 87,5% des cas. CEC: Seulement 8 des répondants (16%) avaient obtenus ou étaient en train de suivre un CEC, essentiellement par intérêt personnel (62,5%) ou pour l'obtention d'un poste d'AHU dans 37,5% des cas. Pour la majorité des résidents ayant passée un CEC (75%), son contenu était jugé utile pour la pratique. Les CEC se répartissaient comme suit: - CEC d'anatomie: 3 cas - CEC de coelioscopie: 3 cas - CEC de maladies inflammatoires chroniques de l'intestin (MICI): 2 cas travail universitaire: La réalisation d´un travail universitaire était jugée bénéfique pour la formation chirurgicale par 34% des répondants. La réalisation d´un travail en tant que premier auteur (présentation au congrès, rédaction d´un article original) était rapportée dans 40% des cas. Les raisons de réaliser un tel travail étaient par ordre croissant l´intérêt personnel (40%), et la carrière (poste d'AHU) (60%).

**Tableau 3:** Formations complémentaires au cours du résidanat

Formation complémentaire		Nombre/Pourcentage
**Stage inter-CHU**	Oui ou en cours	24 (48)
	En projet	4 (8)
	Non	22 (44)
**CEC**	Oui ou en cours	8 (16)
	Non	42 (84)
**Travail universitaire (communication ou article en premier auteur)**	Oui	20 (40)
	Non	30 (60)

**Figure 4:**
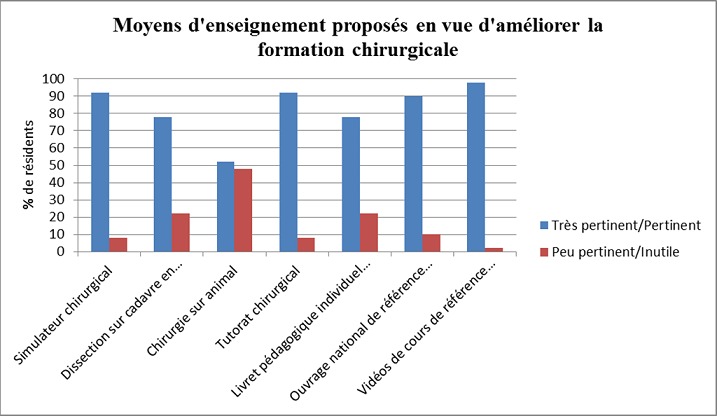
Moyens proposés en vue d'améliorer la formation chirurgicale

**Avis sur la formation actuelle**: Parmi les résidents interrogés, 90% (n = 45) jugeaient que leur formation chirurgicale n´était pas satisfaisante, du fait d´un défaut de formation pratique (13,3%), théorique (11,1%) ou des deux (75,6%). **Evaluation de la formation et perspectives: Evaluation de la formation:** En dehors des examens de fin de spécialité de chirurgie générale et digestive, il n'existe aucune évaluation formative au cours du résidanat aussi bien sur le plan théorique que pratique. 27 (54%) des répondants souhaitaient la mise en place d´une évaluation objective de l´apprentissage chirurgical pratique au cours du résidanat de chirurgie générale et digestive. Selon les résidents désirant l'instauration de cette évaluation pratique au bloc opératoire, le résident à évaluer sera opérateur sur des interventions types aidé d'un résident avec des objectifs chirurgicaux définis par année (Par exemple: 1^ère^ année: acquisition des techniques d'ouverture et fermeture + appendicectomie. 2^ème^ année: acquisitions des interventions simples courantes types vésicule biliaire, hernie. 3^ème^, 4^ème^, et 5^ème^ année: interventions plus complexes types rate, colon, estomac). Cette évaluation peut se faire de façon annuelle par un chirurgien sénior d'un autre centre ou par un professeur (Pr) du service avec avis des Pr. Ag et AHU ou par deux observateurs: un du service et un extérieur du service. L’évaluation porte sur le temps opératoire, le nombre d'incidents per-opératoires avec une note globale de la gestion de l'acte. Perspectives: Les moyens proposés en vue d'améliorer la formation chirurgicale sont décrits dans la Figure 4. L´ensemble des propositions était jugé pertinent, en particulier (>80% d´avis favorables) l´apprentissage sur simulateur chirurgical, l´instauration, l´existence d´un ouvrage national de référence destiné aux résidents et disponible en ligne, l´existence de vidéos de cours de références disponibles en ligne et enfin l´institution d´un tutorat tout au long du résidanat par un chirurgien sénior type AHU ou Pr Ag (65,1%), Pr(29,1%), ou indéterminé (5,8%).

## Discussion

Plus de la moitié des résidents de chirurgie générale et digestive ont répondu à cette enquête qui comporte probablement de nombreux biais (dont le principal est un biais d’échantillonnage), mais présente une cartographie extemporanée de cette population de “jeunes chirurgiens” en formation. Leurs ressentis sur la formation actuelle, leurs attentes éventuelles permettant de se poser certaines questions quant à l'organisation ou la modification de l'enseignement de la chirurgie générale et digestive en Tunisie. Ainsi, les caractéristiques des répondants ne différaient pas des non répondants, mais, il est possible que les résidents ayant une vocation universitaire soient plus enclins à participer spontanément à ce type d´enquête académique [1]. Le changement démographique majeur qualitatif, est celui de la féminisation constante de la médecine ainsi que de la chirurgie. Cette féminisation de la profession chirurgicale a été soulignée par une enquête francaise récente parmi les internes en formation avec un sexe ratio de 1 pour 1[2]. Dans notre étude “ extemporanée ”, le sexe ratio (homme/femme) était de 1,9, mais ne doit pas faire oublier que la spécialité de chirurgie générale et digestive bien que très masculine se féminise progressivement de façon similaire et décalée au tronc commun des études de médecine. On assiste actuellement en Tunisie à une désaffection pour la chirurgie générale et digestive. En effet, dans notre étude 44% des répondants l'ont choisi comme spécialité pour des raisons de classement au concours de résidanat qui empêchait de choisir la spécialité voulue. Une étude réalisée en France en 2008 a analysé les souhaits et les motivations professionnelles de 1742 étudiants en médecine de fin de deuxième cycle pour leur choix de spécialités chirurgicales. 62% des étudiants étaient de sexe féminin et 34% des étudiants voulaient s'orienter vers une spécialité chirurgicale et seulement 10% des étudiants souhaitaient devenir chirurgien digestif [3]. Une autre étude française, portant sur 929 étudiants en médecine et sur les nouveaux internes reçus à l'examen classant national 2008 confirme qu'il existe une désaffection pour la chirurgie générale et digestive, majoritairement induite par un a priori négatif de cette spécialité, notamment concernant la charge de travail [4]. La formation au métier de chirurgien requiert, à la différence de nombreuses disciplines médicales, un enseignement technique nécessaire à l'accomplissement des interventions chirurgicales. En Tunisie, l'enseignement technique est basé essentiellement sur le compagnonnage. Il s'agit d'une formation opératoire, dispensée par un chirurgien sénior à un résident dans des conditions réelles lui permettant de réaliser toute ou une partie d'une intervention chirurgicale sous le contrôle effectif, direct et constant du chirurgien sénior. Ce dernier peut, pas à pas, corriger les gestes ou la stratégie opératoire du résident. Le comportement de type “ chirurgien fantôme” soulève des problèmes éthiques et légaux pour le chirurgien sénior et le résident qui ne seront pas abordés ici. A l'inverse, un travail en binôme avec éventuellement tutorat est l'essence même du compagnonnage qui fait tout l'intérêt de cette méthode d'enseignement. Un élément important est sans doute d'adapter la difficulté de l'intervention chirurgicale à la compétence et l'expérience du résident [5, 6]. Il convient de procéder à une augmentation progressive des difficultés opératoires [7]. Des exposés faits par le résident lui-même, relatifs à la technique opératoire, plus que des cours dispensés par les séniors, améliore les performances chirurgicales [8].

Par ailleurs, le développement de la chirurgie mini-invasive modifie probablement la qualité de l'apprentissage [9]. Le caractère relativement récent de l'essor de ces techniques, qui sont parfois en cours de mise au point, peut rendre moins aisé le fait d'aider un résident en raison d'une moins bonne maîtrise opératoire du chirurgien sénior lui-même. De plus le contrôle de l'activité du résident peut être difficile. Pour l'enseignement de la chirurgie mini-invasive, il convient comme pour la chirurgie conventionnelle de commencer par des interventions simples. Selon Friedman et Pace [10], la cholécystectomie sous laparoscopie peut être enseignée de façon progressive par le compagnonnage sans avoir recours à un cycle de formation spécialisé. Il est donc souhaitable, afin d'améliorer le niveau de l'enseignement, d'inclure dans l’équipe chirurgicale un chirurgien spécialisé en chirurgie mini-invasive ce qui augmente la fréquence de ces interventions et contribue à une meilleure formation des résidents [11]. Cependant, parmi 233 résidents canadiens en chirurgie générale ayant répondu à un questionnaire leur demandant de porter une appréciation sur leur formation en chirurgie mini-invasive, seulement 18% jugent que leur formation est insuffisante [12]. Il est probable que le fait d'aider un résident à réaliser une intervention chirurgicale allonge la durée opératoire [13–17]. Cependant, la répétition de l'intervention permet rapidement au résident de gagner en rapidité dans la chirurgie conventionnelle, comme pour la chirurgie mini-invasive [14]. Aider un résident à opérer entraînerait une augmentation des pertes sanguines [13, 14]. Cependant plusieurs auteurs s'accordent sur le fait que le résultat final ne diffère pas de celui obtenu si le chirurgien sénior avait opéré lui-même [18]. L'efficacité du compagnonnage est difficile voire impossible à évaluer. Elle dépend des personnalités du résident et du chirurgien senior, ainsi que des rapports qu'ils peuvent établir. Les résidents sont de disponibilité, de compétence inégales et leur envie d'apprendre est variable. Les chirurgiens séniors peuvent être plus ou moins brillants ou motivés par l'enseignement. L'efficacité de cette méthode d'enseignement présente donc une variabilité inter-individuelle. Il est probable qu'un résident zélé bénéficiera plus de ce type d'enseignement qu'un résident moins motivé. L'attribution de la connaissance n'est donc pas équitable, avec tendance à l’élitisme. Il est cependant impératif que chaque résident reçoive l'enseignement minimal afin d'en faire un chirurgien compétent. Cet enseignement minimal est difficile à évaluer car dans notre système il n'est pas quantifié ce qui rend impossible sa validation. Enfin, il n'est pas réglementé et peut être perçu, en particulier par les patients, comme une rupture du contrat moral que le chirurgien sénior a lié avec eux. Une étude montre que 32% des patients s'opposent à ce qu'une intervention chirurgicale soit réalisée sur leur personne par un résident dirigé par un chirurgien sénior [19]. Enfin, un incident opératoire ou post-opératoire met en jeu la responsabilité du chirurgien senior.

Comme tout enseignement, la formation technique chirurgicale doit être sanctionnée par une validation des capacités opératoires. A l'heure actuelle en Tunisie, un résident à la fin de son cursus remplit les exigences pour passer le concours de fin de spécialité s'il a effectué des stages validés par les chefs de service. Il n'a pas à rendre compte de son activité opératoire, en opérateur ou premier aide. D'autres pays exigent la présentation d'un livret opératoire du résident justifiant d'un nombre requis de différentes interventions en tant qu'opérateur pour obtenir le diplôme [13, 20, 21]. L’évaluation de l'aptitude pédagogique des chirurgiens seniors par les internes (équivalents des résidents en Tunisie) est appliquée dans certains centres [22]. Plusieurs méthodes d'enseignement de technique opératoire peuvent venir compléter le compagnonnage. La dissection et la chirurgie cadavériques sont des exercices formateurs. Une équipe de neurochirurgie propose aux résidents de participer à l'autopsie de patients décédés dans le service au moyen de techniques microchirurgicales ou endoscopiques [23]. La même équipe a bien montré que l'entraînement opératoire sur le cadavre permet d'améliorer les performances opératoires in vivo [23]. Cependant, même si le cadavre est frais, les conditions opératoires ne sont bien sûr pas les mêmes que sur un organisme vivant. En Tunisie, ce mode d'enseignement se heurte à un problème légal. La chirurgie sur l'animal est un exercice plus proche de la réalité [24, 25]. Son but est de permettre la réalisation d'interventions chirurgicales dans des conditions d'autonomie opératoire. De plus, ceci stimule la réflexion sur la gestuelle et les stratégies opératoires. L'idéal est de regrouper les résidents en binômes de niveau proches, avec un encadrement par un sénior. Les deux modalités pédagogiques précédentes peuvent être optimisées par un enregistrement vidéo de l'intervention en cours, qui retransmise dans une salle voisine, autorise des commentaires des enseignants et des enseignés. De plus, l'auto-observation différée, surtout si elle est complétée par les commentaires d'un sénior permet une amélioration des performances opératoires [25, 26]. Le développement de l'informatique et de la numérisation permet de proposer aux résidents des moyens d'entraînement opératoire de type chirurgie virtuelle [27, 28]. La perception des trois dimensions de l'espace est fondamentale, en particulier en chirurgie mini-invasive pendant laquelle le chirurgien visualise le site opératoire sur un écran vidéo, et peut être favorisée par la chirurgie virtuelle sur ordinateur [29]. Le développement de la chirurgie assistée par l'ordinateur est un argument supplémentaire pour développer ce genre de logiciels utilisables tant lors d'entraînement que lors d'interventions réelles. Durant l'année universitaire 2008-2009, l’école de chirurgie de Paris a mis en place un nouveau programme de perfectionnement à la laparoscopie, destiné aux chirurgiens en formation, afin de leur fournir les bases techniques indispensables selon un schéma cadré et progressif [30]. L'enseignement de la chirurgie peut être complété par un apprentissage utilisant les différentes techniques de simulation. L'apprentissage par simulation, largement répandu outre-atlantique, permet, sans aucun risque pour les patients, d'intervenir sur plusieurs éléments de la formation chirurgicale. Enfin, l’évolution des pratiques chirurgicales doit intégrer une dimension médicolégale et la notion de certification et de formation continue. Ainsi, outre-atlantique, l'impact médicolégal et économique d'une formation sur simulateur avant certification pour certaines spécialités ou techniques “ à risque” est maintenant reconnu [31]. Les patients sont rassurés que les praticiens qui s'apprêtent à pratiquer des actes invasifs sur eux aient été formés sur simulateurs. Pour Hamilton et al, la simulation pré-opératoire d'une cure de hernie par laparoscopie améliore les performances opératoires [32].

La chirurgie étant un métier artisanal fondé sur une exécution personnelle, l'apprentissage du geste chirurgical lui-même est la source d'angoisse professionnelle prenant le pas sur le reste de la formation. En effet, le geste et la technique chirurgicale pure ne demeure qu'une partie de la prise en charge globale du malade qui est, souvent négligée par les résidents en formation [33]. L'impression globale de cette enquête est que les résidents ont toujours l'impression d’être “mal formés” ou “pourraient être mieux formés”, notamment lorsqu'on les laisse peu opérer. En effet, sur l´ensemble de la population interrogée, 90% (n = 45) jugeaient que leur formation chirurgicale n´était pas satisfaisante, du fait d´un défaut de formation pratique (13,3%), théorique (11,1%) ou des deux (75,6%). Paradoxalement les résidents en chirurgie Tunisiens sont parmi les “ jeunes chirurgiens ” dans le monde étant le plus tôt dans leur cursus, et le plus intensément plongé dans “ l'acte” et la pratique chirurgicale, particularité singulière de notre système partout envié et plébiscité par nos confrères étrangers et, selon eux conférant à l’école tunisienne sa réputation de haute technicité. La pression Médico-légale est sans cesse grandissante et dont l'impact sur le mode d'enseignement de la chirurgie retentit de façon proportionnelle sur la majorité des systèmes de soins fonctionnant “ à l'occidentale“ avec cependant des différences notables d'un pays à l'autre. Dans notre étude pouvons-nous constater que 62% des répondants jugeaient qu'il n´existait pas de politique visant à laisser opérer les résidents, et que le manque de temps et le désir du sénior de prendre en charge lui-même le malade étaient majoritairement évoqués (dans respectivement 68% et 78%) tandis que la pression médico-légale ne l’était que rarement. Il nous apparaît par ailleurs nécessaire de pondérer ce résultat par le fait qu'il ne représente que l'impression “ressentie” des jeunes chirurgiens avec un biais potentiel qui résiderait dans une mésinterprétation potentielle des réelles causes à l'origine de cette politique “ne laissant pas opérer ”. Afin de répondre plus justement à cette question il faudrait réaliser en parallèle une étude portant sur les “ enseignants ” et confronter les résultats avec ceux des “ enseignés ”. Concernant les modalités d'enseignement, plusieurs propositions nous paraissent exister déjà ou à développer: La première et globalement la plus simple est d'informer les résidents des ressources à leur disposition déjà existantes et souvent de façon aisée. Nombre d'ouvrages de références existent allant de l'encyclopédie chirurgicale ou ses équivalents pour la technique en passant par les recommandations factuelles et révisées par les sociétés savantes chirurgicales pour le coté théorique. De façon similaire, complémentaire et plus moderne, il existe déjà de nombreux sites internet mettant à dispositions de l'information littéraire, vidéo ou interactive pouvant répondre à déjà beaucoup des attentes des résidents en formation. Concernant l'aspect pratique, la mise à disposition de certains lieux voir “ écoles de formations ” chirurgicales publiques ou privées telles que l’école de chirurgie de l'assistance publique des hôpitaux de Paris (AP-HP) à Paris ou certains Training Center (Souvent dirigés ou financés par les firmes industrielles) peuvent répondre, tout du moins partiellement, à la demande de formation pratique par dissection sur cadavre, animaux ou de plus en plus par simulation informatique ou robotisée. Cette dernière actuellement en pleine expansion notamment dans les pays anglo-saxons ou du Nord a montré son impact sur à la fois la réduction du temps global de formation technique initiale mais également sur la sécurité et l'effectivité des soins dans plusieurs publications [34–38]. En Tunisie, ses moyens sont actuellement indisponibles de par leur coût initial d'investissement mais pourraient constituer une alternative dans l'avenir. L'une des solutions serait le développement de partenariats entre les industriels de santé et les tutelles en charge de la formation initiale et garante de sa qualité.

## Conclusion

La majorité des résidents de chirurgie générale et digestive jugent leur formation non satisfaisante. Une meilleure information sur les ressources déjà existantes, un accès à un apprentissage sur simulateur chirurgical et un renforcement du compagnonnage, permettraient de diminuer ce sentiment d'insatisfaction. Il faudra probablement mener une véritable politique incitative, donner les moyens à la chirurgie d’être une profession revalorisée dont la pénibilité est enfin reconnue et de faire évoluer l'image et la réputation de cette spécialité en tenant compte notamment des nouvelles aspirations des jeunes médecins et de la féminisation de la profession médicale. L'enjeu est d'importance car c'est à ce prix que les jeunes reviendront vers la chirurgie et seront des chirurgiens compétents, dévoués et volontaires.
